# STNet: shape and texture joint learning through two-stream network for knowledge-guided image recognition

**DOI:** 10.3389/fnins.2023.1212049

**Published:** 2023-06-15

**Authors:** Xijing Wang, Hongcheng Han, Mengrui Xu, Shengpeng Li, Dong Zhang, Shaoyi Du, Meifeng Xu

**Affiliations:** ^1^National Key Laboratory of Human-Machine Hybrid Augmented Intelligence, National Engineering Research Center for Visual Information and Applications, Institute of Artificial Intelligence and Robotics, Xi'an Jiaotong University, Xi'an, China; ^2^The School of Software Engineering, Xi'an Jiaotong University, Xi'an, China; ^3^The School of Automation Science and Engineering, Xi'an Jiaotong University, Xi'an, China; ^4^The Second Affiliated Hospital of Xi'an Jiaotong University (Xibei Hospital), Xi'an, China

**Keywords:** computer-aided diagnosis, image recognition, feature fusion, joint learning, two-stream network, brain-like information processing

## Abstract

**Introduction:**

The human brain processes shape and texture information separately through different neurons in the visual system. In intelligent computer-aided imaging diagnosis, pre-trained feature extractors are commonly used in various medical image recognition methods, common pre-training datasets such as ImageNet tend to improve the texture representation of the model but make it ignore many shape features. Weak shape feature representation is disadvantageous for some tasks that focus on shape features in medical image analysis.

**Methods:**

Inspired by the function of neurons in the human brain, in this paper, we proposed a shape-and-texture-biased two-stream network to enhance the shape feature representation in knowledge-guided medical image analysis. First, the two-stream network shape-biased stream and a texture-biased stream are constructed through classification and segmentation multi-task joint learning. Second, we propose pyramid-grouped convolution to enhance the texture feature representation and introduce deformable convolution to enhance the shape feature extraction. Third, we used a channel-attention-based feature selection module in shape and texture feature fusion to focus on the key features and eliminate information redundancy caused by feature fusion. Finally, aiming at the problem of model optimization difficulty caused by the imbalance in the number of benign and malignant samples in medical images, an asymmetric loss function was introduced to improve the robustness of the model.

**Results and conclusion:**

We applied our method to the melanoma recognition task on ISIC-2019 and XJTU-MM datasets, which focus on both the texture and shape of the lesions. The experimental results on dermoscopic image recognition and pathological image recognition datasets show the proposed method outperforms the compared algorithms and prove the effectiveness of our method.

## 1. Introduction

Computer-aided diagnosis (CAD) has been a research hotspot for the past few decades. CAD automatically analyzes the patient data through machine learning methods to make an assessment of the patient's condition (Yanase and Triantaphyllou, 2019; Chan et al., 2020). Medical image analysis is one of the most important fields in CAD technologies, it helps read imaging data to improve the diagnosis efficiency. An intelligent medical image analysis model can share the workload of radiologists and pathologists, and enables areas with underdeveloped medical resources to achieve high-level imaging analysis at low cost (Shen et al., 2017; Kurc et al., 2020).

In the past decade, medical image analysis methods have grown by leaps and bounds due to the development of deep learning and computer vision algorithms. Powerful feature representation ability enables deep neural networks to learn complex hidden features from a large amount of training data, which overcomes the difficulty of manual feature design in traditional medical image analysis methods. However, there are still challenges to be addressed in current deep learning-based algorithms for medical image analysis, with weak shape representation being one of the most critical issues. On the one hand, in the commonly used convolutional neural network (CNN), the limited receptive field of kernels tends to fit local features during kernel parameter learning. Although the range of the receptive field of deep convolutional kernels on original images gradually increases as layers deepen, deeper layers weaken their connection with original images, which limits networks in modeling shape features at larger scales (Luo et al., 2016; Araujo et al., 2019). On the other hand, pre-trained parameters are frequently employed in medical image recognition techniques to expedite convergence during training and potentially enhance model performance. Given the paucity of annotated data in medical images, large-scale natural image datasets such as ImageNet (Deng et al., 2009; Russakovsky et al., 2015) are commonly utilized as pre-training datasets. However, the research of Geirhos et al. (2018) indicates that the deep neural network pre-trained on ImageNet is biased to focus on the texture features and has relatively weak shape feature representation ability.

**Figure 1:**
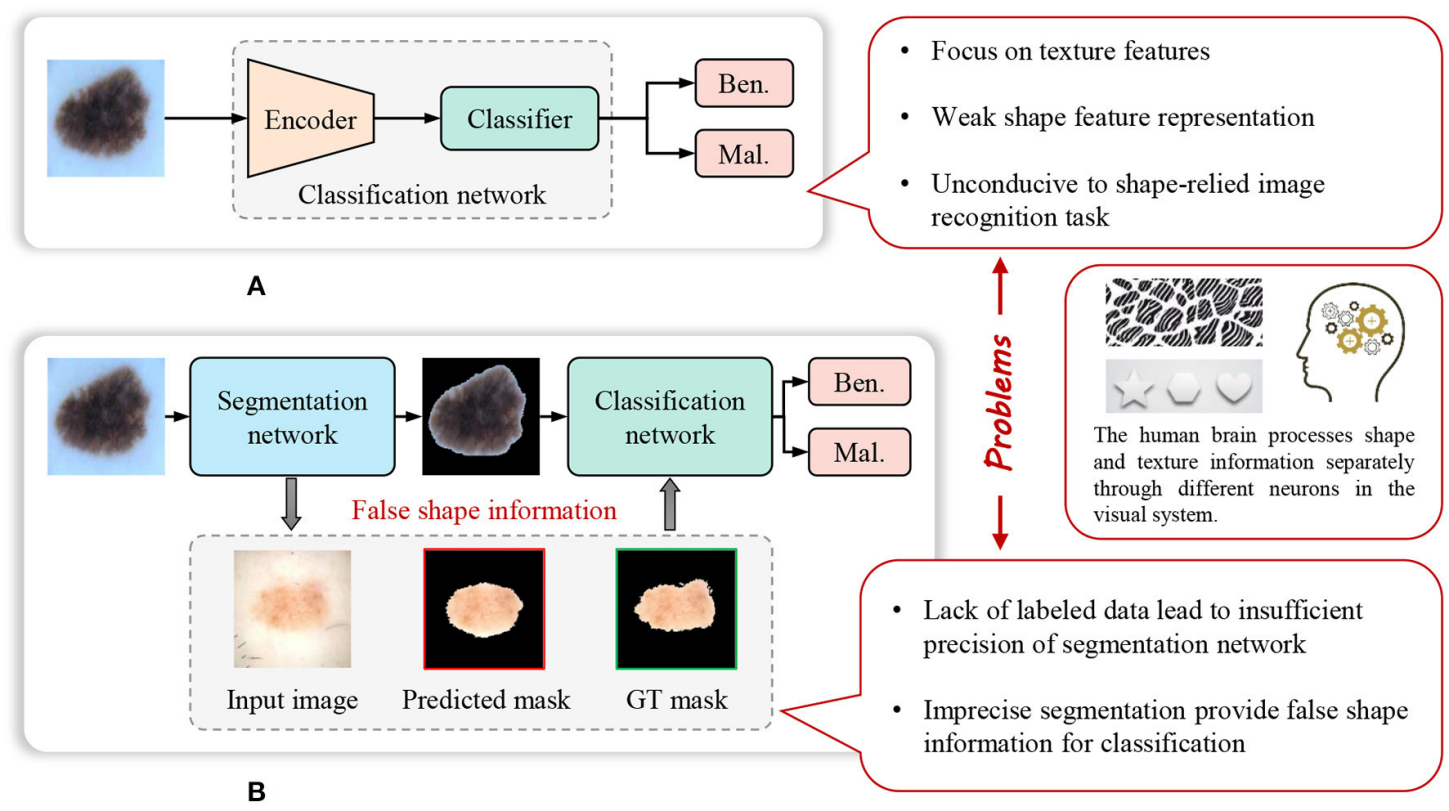
Weak feature representation problem of many existing methods for image recognition in computer-aided diagnosis. **(A)** Common image recognition model. **(B)** Cascade segmentation and classification model.

The weak representation of shapes, caused by the limitations of the model and pre-training datasets, significantly impacts the performance of the model on certain shape-dependent medical image tasks. As, Figure 1 shows, cascade segmentation and classification model (Chang, 2017) can solve the problem in some scenarios, it uses a segmentation network to obtain the mask of a lesion, and then use the segmented lesion image as the input of the classification network, providing shape information for classification, eliminating the background noise. However, the lack of sufficient training data is a prevalent issue in various medical image analysis tasks, resulting in inadequate precision of the trained segmentation task. Inaccurate segmentation can provide erroneous shape information for classification. In addition, the cascade segmentation and classification model contains two encoders and one decoder, and they are cascaded, the research of He et al. (2017) indicates that repetitive encoding and decoding operations yield minimal improvements to the quality of extracted features.

In order to solve the above problems, we proposed a shape-and-texture-biased two-stream network to enhance the shape feature representation in knowledge-guided medical image analysis. The human brain processes shape and texture information separately through different neurons in the visual system, inspired by that, first, the two-stream network shape-biased stream and a texture-biased stream are constructed through classification and segmentation multi-task joint learning. Second, we propose pyramid-grouped convolution (PGC) to enhance the texture feature representation, and introduce deformable convolution (DC) to enhance the shape feature extraction. Third, we used a channel-attention-based feature selection module in shape and texture feature fusion to focus on the key features and eliminate information redundancy caused by feature fusion. Finally, aiming at the problem of model optimization difficulty caused by the imbalance in the number of benign and malignant samples in medical images, an asymmetric loss function was introduced to improve the robustness of the model. We applied our method to the melanoma recognition task on ISIC-2019 (Rotemberg et al., 2021) and XJTU-MM datasets, which focuses on both the texture and shape of the lesions. The experimental results on dermatoscopic image recognition and pathological image recognition show that the proposed method outperforms the compared algorithms and prove the effectiveness of our method.

The main contributions of this work are enumerated as follows:

- We propose the shape and texture joint learning two-stream network for knowledge-guided medical image recognition, taking into account the learning of shape features and texture features by the network, addressing the weak shape representation problem of existed methods.
- We propose pyramid-grouped convolution to enhance the texture feature representation, and introduce deformable convolution to address the limitation of fixed respective fields, enhancing the shape feature extraction.
- We construct the shape and texture fusion module based on channel attention mechanism to focus on the essential features and eliminate the noise, reducing the information redundancy caused by feature fusion.
- We introduce the asymmetric loss function for optimization, reducing the impact of commonly existed sample imbalance problem in medical image datasets.

## 2. Related work

### 2.1. Knowledge-guided medical image analysis

Most of the key technologies in medical image analysis come from general computer vision algorithms, however, the image characteristics and the data distribution are different between natural images and medical images. Constructing appropriate deep neural network model with the guidance of the prior knowledge from pathology and radiology is important for improving model performance in specific medical analysis tasks.

Fan et al. (2017) proposed a novel automatic segmentation algorithm using saliency combined with Otsu threshold for dermoscopy images, which extracted prior information on healthy skin to construct the color saliency map and brightness saliency map respectively. Ahn et al. (2017) proposed a saliency-based lesion segmentation method in dermoscopic images, using the reconstruction errors derived from a sparse representation model coupled with a novel background detection. Yang et al. (2023) proposed a Multi-scale Fully-shared Fusion Network (MFF-Net) that gathers features of dermoscopic images and clinical images for skin lesion classification. Zhang et al. (2018a) used deep learning algorithms to help diagnose four common cutaneous diseases based on dermoscopic images and summarized classification/diagnosis scenarios based on domain expert knowledge and semantically represented them in a hierarchical structure to improve the accuracy of the algorithm. Clinical prior knowledge is also widely applied to the analysis of ultrasound images and other medical images. Liu et al. (2019b) proposed a novel deep-learning-based CAD system, guided by task-specific prior knowledge, for automated nodule detection and classification in ultrasound images. Chen et al. (2021) proposed a knowledge-guided data augmentation framework for breast lesion classification, which consists of a modal translater and a semantic inverter, achieving cross-modal and semantic data augmentation simultaneously. Shi et al. (2020) proposed a knowledge-guided synthetic medical image adversarial augmentation method for ultrasonography thyroid nodule classification, extracting domain knowledge from standardized terminology to improve the classification performance. Yang et al. (2021) proposed a multi-task cascade deep learning model (MCDLM), which integrates radiologists' various domain knowledge (DK) and used multimodal ultrasound images for automatic diagnosis of thyroid nodules. Han et al. (2020) proposed an ensemble learning method for panoramic radiographs recognition based on the characteristics of each stage of tooth growth. Ni et al. (2013) proposed a novel learning-based automatic method to detect the fetal head for the measurement of head circumference from ultrasound images and used prior knowledge and online imaging parameters to guide the sliding window-based head detection. Pan et al. (2022) proposed a two-stage network with prior knowledge guidance for medullary thyroid carcinoma recognition in ultrasound images. Meanwhile, extracting and fusing semantic features of solid tissues and calcification for better recognizing the segmented nodules. Zhou et al. (2022) proposed a rheumatoid arthritis knowledge-guided (RATING) system for scoring rheumatoid arthritis activity from multimodal ultrasound images, leveraging diagnostic paradigm and experience to enhance the robustness. Lu et al. (2023) proposed a Prior Knowledge-based Relation Transformer Network (PKRT-Net), which employed the clinical prior knowledge to assist OC segmentation. Gao et al. (2021) proposed a medical-knowledge-guided one-class classification approach that leverages domain-specific knowledge of classification tasks to boost the model's performance and showed superior model performance on three different clinical image classification tasks. Zhang et al. (2023) proposed coarse-to-fine method for melanoma and nevi recognition according to distribution of inter-class and intra-class differences as summarized by dermatologists.

Prior knowledge provides inspiration for medical image analysis design, in this paper, we innovate a novel method for shape-relied medical image recognition.

### 2.2. Shape and texture feature fusion

Aiming at the problem of weak shape representation of existing CNN-based medical image recognition models, we investigate the texture and shape feature fusion algorithms designed for various tasks.

Al-Osaimi et al. (2011) proposed spatially optimized data/pixel-level fusion of 3-D shape and texture for face recognition. Lu et al. (2017) proposed a face image retrieval method based on shape and texture feature fusion, which used accurate facial landmark locations as shape features and utilized shape priors to provide discriminative texture features. Kotsia et al. (2008) proposed a novel method based on the fusion of texture and shape information for facial expression and Facial Action Unit (FAU) recognition from video sequences and used various approaches to perform texture and shape feature fusion, among which were SVMs and Median Radial Basis Functions (MRBFs). Anantharatnasamy et al. (2013) proposed a content-based image retrieval system based on three major types of visual information including color, texture, shape, and their distances to the origin in a three dimensional space for the retrieval. Sumathi and Kumar (2012) extracted edge and texture features using Gabor filter and fused them for plant leaf classification. Xiong et al. (2007) proposed a Statistical Shape and Radio texture fusion model for facial expression sequence synthesis, processing facial shape and texture separately and fusing them together to synthesize the final result. Jo et al. (2014) proposed a new method for eye state classification to detect diver drowsiness, which extracted and fused features from both eyes. Zhang et al. (2020) proposed two-stream networks to enhance the extraction of shape and texture respectively for clothing classification and attribute recognition.

These researches use various of methods to enhance the texture and shape feature learning on specific data. For shape-relied medical image recognition tasks, we design the model to realize that with the guidance of the prior knowledge, such as visual characteristics and category distribution.

## 3. Methodology

### 3.1. Framework

**Figure 2:**
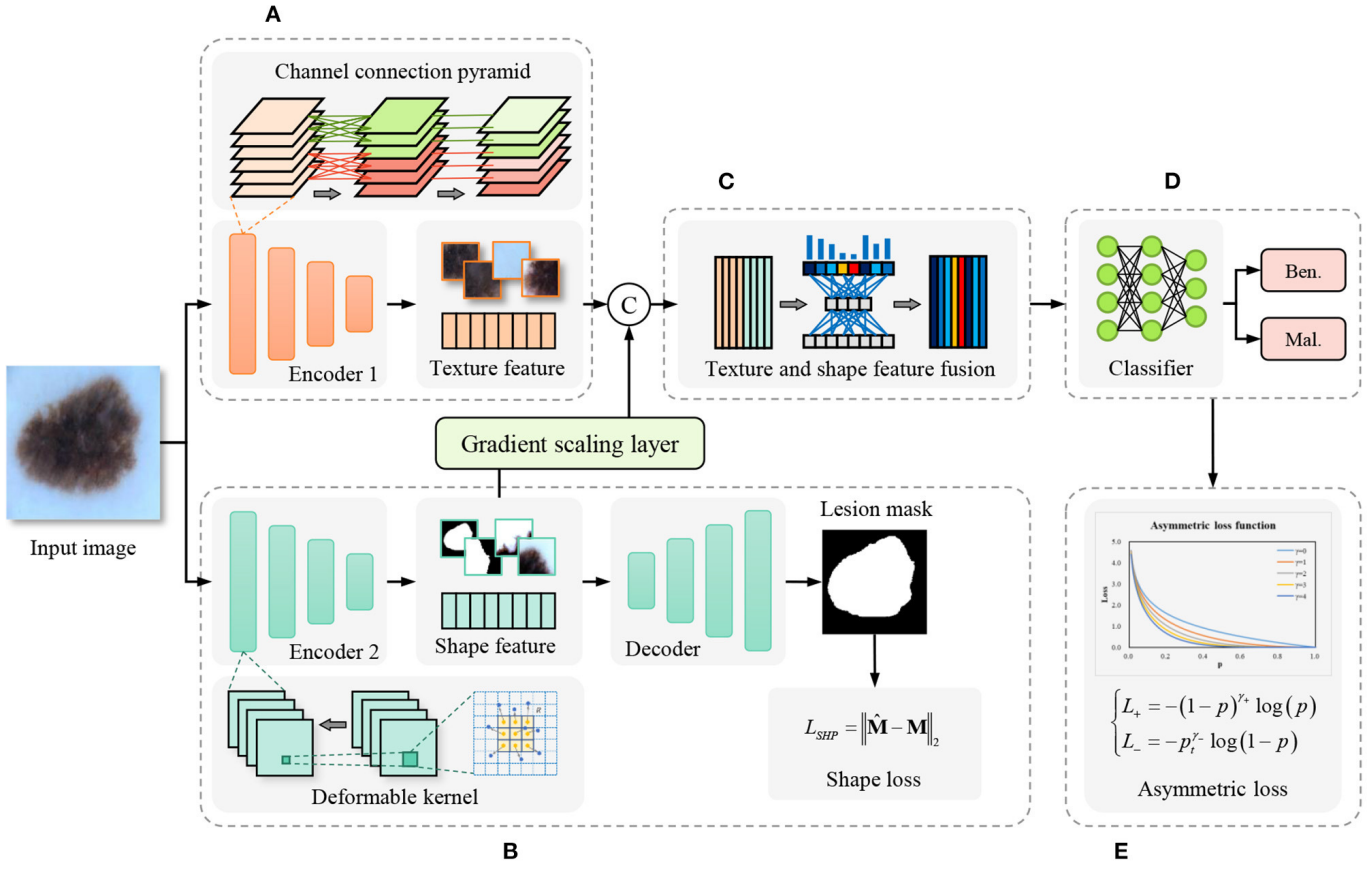
Framework of the proposed shape and texture joint learning two-stream network. **(A)** Texture-biased stream. **(B)** Shape-biased stream. **(C)** Feature fusion module. **(D)** Classifier. **(E)** Asymmetric loss.

In contrast to the cascade segmentation and classification model, our proposed model employs a two-stream network for joint learning of shape and texture, mitigating the impact of imprecise segmentation on shape information in the former. The overall framework of the proposed method is shown as Figure 2, the input image is fed into the parallel texture-biased stream and shape-biased stream. First, the texture-biased stream consists of a feature encoder, which is pre-trained on texture-biased large-scale dataset, such as ImageNet. To further enhance the texture feature representation ability of the texture feature encoder, we reconstruct the convolutional block using the proposed channel connection pyramid mechanism. Second, the shape-biased stream contains an encoder-decoder based network, the encoder extracts the shape features and the decoder generates the lesion mask, the quality of the extracted shape features is supervised by *L*2 loss function between the predicted mask and the ground truth mask. Third, the texture feature and the shape feature are concatenated and input to the feature fusion module, to address the information redundancy problem in feature fusion, we construct the feature fusion module based on channel attention mechanism to focus on the essential features and eliminate the effects of noise. In addition, to balance the texture-biased learning and shape-biased learning, the gradient scaling layer is added between the shape feature map and the concatenation operation to weight the gradient in the back propagation. Then, the fully connected layer classifier is used to output the classification results. Finally, to overcome the optimization difficulty caused by the problem of imbalanced samples in medical image datasets, we introduce the asymmetric loss to enhance the attention of the model to the categories with smaller numbers of samples.

### 3.2. Texture-biased stream

The texture-biased stream is constructed by the texture feature encoder pre-trained on texture-biased dataset ImageNet. To enhance the texture feature representation, we improve the channel connections in convolutional blocks. In the standard convolution operation, each kernel is connected to every channel of the input feature map. However, while the large number of learnable parameters provides a powerful fitting ability for the network, overly dense connections can lead to significant information redundancy and unnecessary computational burden (Huang et al., 2017; Ma et al., 2018; Zhang et al., 2018b). Grouped convolution mechanism (Xie et al., 2017; Zhang H. et al., 2022) provides an efficient way to solve the problem, it divides the input feature map into several groups in the channel dimension, each kernel has connections to the specific group only rather than all channels of the input feature map. With the same number of output feature map channels, channel-wise connections become sparser, thereby enhancing diagonal correlations between channels. Depth-wise convolution (Chollet, 2017) even makes the connections more sparse, which regards each channel of the input feature map as one group to perform grouped convolution. With fewer learnable kernel parameters, depth-wise convolution even shows stronger low-level texture feature representation ability (Guo et al., 2019; Tan and Le, 2019). However, grouped convolution and depth-wise convolution still have problems in balancing the learning of low-level and high-level texture features.

**Figure 3:**
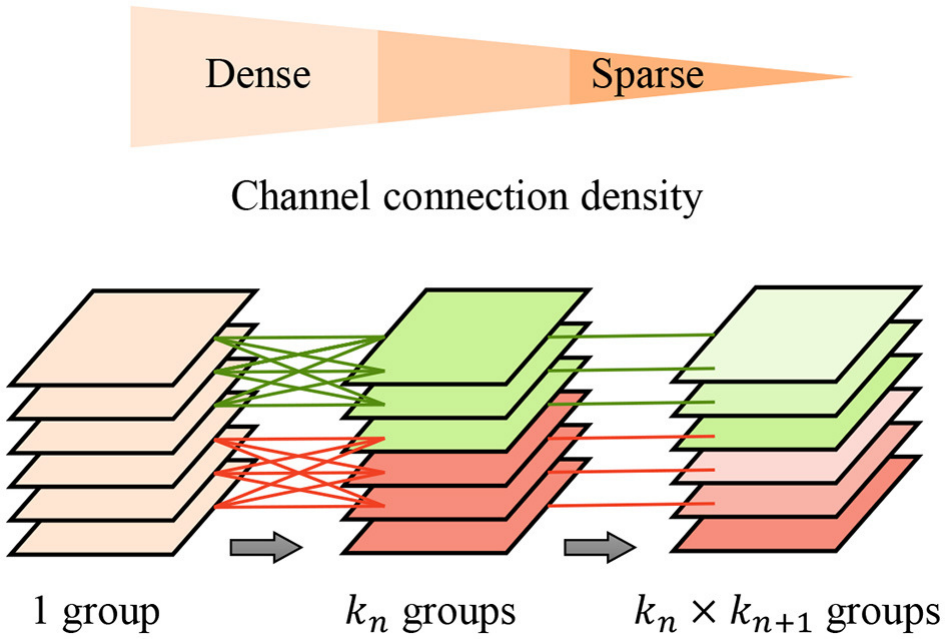
Pyramid-grouped convolution. In each pyramid, the density of channel connection changes layer by layer, and from dense to sparse.

To further improve the feature extraction quality and efficiency, we propose the pyramid-grouped convolution(PGC) mechanism to enhance the feature representation of the texture-biased stream. As Figure 3 shows, In each pyramid-convolutional block, the density of channel connections varies layer by layer, transitioning from dense to sparse. This results in a transition of the channel-wise receptive field of each kernel from large to small, leading to sparser feature encoding compared to conventional grouped convolution and more appropriate channel-wise receptive fields than depth-wise convolution. The PGC blocks are embedded in the backbone network to construct feature encoder of texture-biased stream, enhancing the texture feature representation.

### 3.3. Shape-biased stream

Pixel-wise semantic segmentation model is a learning paradigm conducive to modeling shape features (Long et al., 2015; Guo et al., 2018). In the proposed method, the shape-biased stream is constructed using an encoder-decoder based segmentation network, the decoder generates the lesion mask based on the features extracted from the input image. With the supervision of the *L*2 loss between the predicted mask and the ground truth mask, the encoder is encouraged to learn the shape-biased features. Many encoder-decoder based semantic segmentation models add shortcut connections between encoders and decoders to enhance the contributions of low-level features extracted by shallow layers in encoders to mask generation, which are usually called U-shape networks (Ronneberger et al., 2015; Oktay et al., 2018; Zhou et al., 2018; Zhang et al., 2021). But in the shape-biased stream of our method, all we need is to improve the shape feature representation of the feature map extracted by feature encoder, all the information flow is expected to pass through the deepest feature map, so we did not add any shortcut connection between the encoder and the decoder.

**Figure 4:**
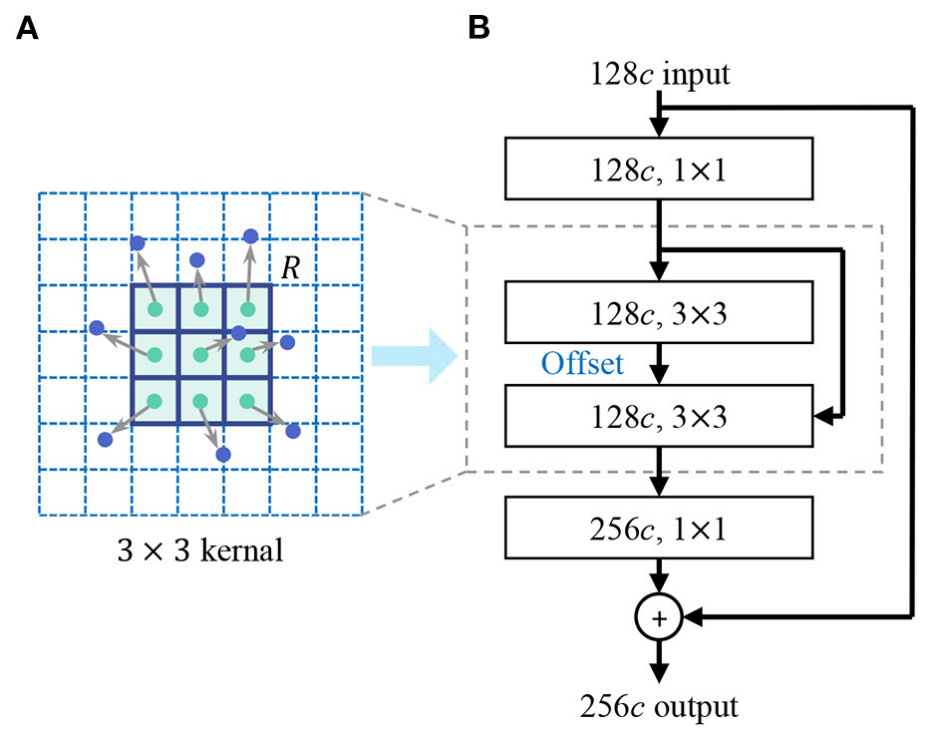
Deformable convolution. **(A)** Deformable kernel. **(B)** Deformable convolutional layer. An offset layer is inserted to learn the offset to transform the rectangular kernel to a kernel with an irregular shape that better match the extracted features. The feature map in the deformable receptive field is resampled through bilinear interpolation according to the parameters of the learned offset.

In the design of the shape encoder network, we introduce the deformable kernel to address the limitation of the rectangular receptive field of the convolution kernel. Irregular-shaped visual features are common in lesion images, for example, the irregular-shape boundary of the lesion in dermoscopic images (Celebi et al., 2019), the irregular-shaped cells in pathological images (Zhang D. et al., 2022). Rectangular convolutional kernels have limitation in extracting these features, especially in extracting low-level shape features. As Figure 4 shows, the discrete feature map is regarded as a continuous two-dimensional distribution, we insert an offset layer to learn a offset to transform the rectangular kernel to an kernel with irregular shape that better match the extracted features. The feature map in the deformable receptive field is resampled through bilinear interpolation according to the parameters of the learned offset. deformable convolution is calculated by


(1) $$\begin{array}{l} y\left(p\right)=\underset{p_{k}\in R}{\sum }w\left(p_{k}\right)\cdot x\left(p+p_{k}+\text{Δ}p_{k}\right), \end{array}$$

where ***y***(*p*) indicates the feature obtained by the convolution on one sampling point *p* of the feature map. ***R*** is the receptive field size of the regular kernel. *p*_*k*_ donates the difference between the sampling points and ***y***(*p*), *k* = 1, 2, 3...*N, N* = |***R***|, Δ*p*_*k*_ is the learned offset, and ***w*** is the kernel parameter. We reconstruct the backbone network of feature encoder using deformable convolution layers, enhancing the representation of irregular-shaped features.

### 3.4. Channel-attention-based texture and shape feature fusion

The feature maps extracted from the texture-biased and shape-biased streams are concatenated to fuse texture and shape features, which expands the scope of the extracted features. However, this also results in a certain degree of information redundancy. Some irrelevant features not only fail to contribute to improving model performance but also increase the risk of overfitting and negatively impact model robustness. To select essential features for lesion recognition and eliminate irrelevant features and noise, we design the texture and shape feature fusion module based on channel attention mechanism.

**Figure 5:**
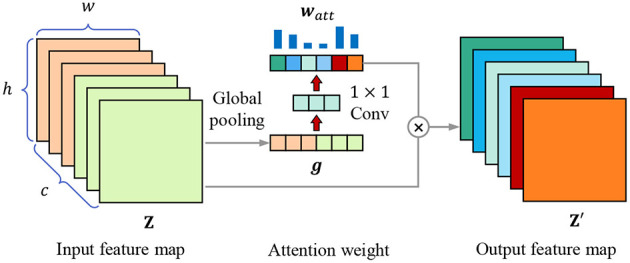
Channel attention mechanism. The attention weight vector ***w***_*att*_ is calculated through global pooling and 1 × 1 convolutional layers, then the input feature map Z is weighted to obtain the output feature map Z′.

Each kernel represents a specific hidden feature, having a specific correlation with lesion recognition, feature selection is equivalent to kernel selection, which can also be regarded as the selection of channels of feature map. We introduce the channel attention mechanism to highlight the essential channels and suppress noise through learning the channel weights based on the global representation of each channel. As Figure 5 shows, for the *w*×*h*×*c* input feature map **Z**, it is first transformed into a 1 × 1 × *c* feature vector ***g*** through global pooling, which combines average pooling and max pooling to balance average and peak characterization, calculating by


(2) $$\begin{array}{l} g_{k}=\frac{1}{2}\left(\frac{1}{wh}\overset{h}{\underset{i=1}{\sum }}\overset{w}{\underset{j=1}{\sum }}z_{i,j,k}+\underset{i,j}{\max}\left(z_{i,j,k}\right)\right), \end{array}$$

where *g*_*k*_ is the element in feature vector ***g***, *z*_*i,j,k*_ is the element in *k*-th channel of feature map **Z**. Then we use two 1 × 1 convolutional layers to obtain the attention weight of each channel, calculating through


(3) $$\begin{array}{l} w_{att}=\delta \left(w_{Conv2}^{T}\cdot \delta \left(w_{Conv1}^{T}\cdot g\right)\right), \end{array}$$

where ***w***_*Conv*1_ and ***w***_*Conv*2_ are the weight parameters of two 1 × 1 convolutional layers, δ(·) is the sigmoid activation function. Finally, the original input feature map is weighted by the weight vector,


(4) $$\begin{array}{l} {\text{Z}}^{\prime }=w_{att}⊗\text{Z}, \end{array}$$

where ⊗ means to multiply ***w***_*att*_ and **Z** channel by channel.

In optimization, the channels that are highly relevant to lesion recognition are highlighted, which eliminates the information redundancy caused by the feature fusion of texture-biased stream and shape-biased stream, and selects the features conductive to lesion recognition, improving the robustness of the model.

### 3.5. Joint learning loss function and optimization

Due to the characteristics of the disease, training data often contains more benign lesions than malignant ones, resulting in insufficient attention given to malignant samples during network training and negatively impacting model optimization (Liu et al., 2019a) and (Xu et al., 2020). If the number of benign samples is forcibly reduced to balance the number of benign and malignant samples, it will lead to insufficient training data.

To address the problem of sample imbalance, we design the asymmetric loss function for medical image recognition with a large amount of negative samples and few positive samples. Different from the commonly used cross-entropy loss shown in Equation (5),


(5) $$\begin{array}{l} L_{CE}=-y\log\left(p\right)-\left(1-y\right)\log\left(1-p\right), \end{array}$$

where *y*_ ∈ _{0, 1} means the ground truth label of the sample, *p* ∈ (0, 1) is the predicted score, when *p*>0.5, the sample is predicted as the positive category, the asymmetric loss decouples the loss of positive and negative categories, reducing the impact of sample imbalance through asymmetric focusing and asymmetric probability transfer, for each sample, the new loss function for classification $L_{CLS}$ is calculated through


(6) $$\begin{array}{l} L_{CLS}=-y{\left(1-p\right)}^{\gamma_{+}}\log\left(p\right)-\left(1-y\right)p^{\gamma_{-}}\log\left(1-p\right), \end{array}$$

where γ_+_ and γ_−_ are the exponential decay factors, the larger the value of the decay factor, the greater the attenuation effect. The adaptive weight factors ${\left(1-p\right)}^{\gamma_{+}}$ and $p^{\gamma_{-}}$ are added to original cross-entropy loss function to asymmetrically scale the loss of positive samples and negative samples, which is better for the optimization in the case of unbalanced samples. We set γ_+_ < γ_−_ to reduce the gradient of the negative samples, strengthening the attention of the model optimization to the positive samples.

In addition, with typical characteristics, some negative samples are easy to identify, to constrain the model to focus on hard samples, we add the probability transfer to the loss function, directly discarding samples which have a low predicted *p* value. The weight factor of $L_{-}$ is reconstructed with the transfer probability *p*_*t*_, which is calculated by


(7) $$\begin{array}{l} p_{t}=\max\left(p-\varphi ,0\right), \end{array}$$

where φ is the probability cutoff threshold, when the predicted *p* is lower than μ, *p*_*t*_ is set to 0. The final asymmetric classification loss function is


(8) $$\begin{array}{l} L_{CLS}=-y{\left(1-p\right)}^{\gamma_{+}}\log\left(p\right)-\left(1-y\right)p_{t}^{\gamma_{-}}\log\left(1-p\right), \end{array}$$

which enables the model to overcome the imbalance of samples in training, and focus on the difficult samples near the discrimination interface, enhancing the robustness of the trained model.

In the optimization of the shape-biased stream, we use *L*2 loss, which is the pixel-wise mean square error between the predicted mask $\hat{\text{M}}$ and the ground truth mask **M**, the shape loss $L_{SHP}$ is


(9) $$\begin{array}{l} L_{SHP}=||\hat{\text{M}}-\text{M}|{|}_{2}, \end{array}$$

In joint learning, texture feature encoder parameter $\theta_{TE}^{*}$ is supervised by *L*_*CLS*_, shape feature decoder parameter $\theta_{SD}^{*}$ is supervised by *L*_*SHP*_, shape feature encoder parameter $\theta_{SE}^{*}$ is supervised by *L*_*CLS*_ and *L*_*SHP*_ to encourage learning shape features that are conductive to lesion classification. In summary, they are optimized by


(10) $$\begin{array}{l} \theta_{TE}^{*}=\underset{\theta_{TE}}{arg min}L_{CLS} \end{array}$$

(11) $$\begin{array}{l} \theta_{SE}^{*}=\underset{\theta_{SE}}{arg min}\left(\alpha L_{CLS}+\beta L_{SHP}\right) \end{array}$$

(12) $$\begin{array}{l} \theta_{SD}^{*}=\underset{\theta_{SD}}{arg min}L_{SHP} \end{array}$$

where α and β is the scaling coefficient to balance $L_{CLS}$ and $L_{SHP}$, which is realized through the gradient scaling layer. Through the cooperative optimization of each module, the proposed method realizes texture and shape joint learning, improving the performance on shape-relied medical image recognition tasks.

## 4. Experiments

### 4.1. Experimental setup

#### 4.1.1. Data preparation

We use two medical image datasets to verify the effectiveness of the proposed method.

- **ISIC-2019:** A public and commonly used dermoscopic image dataset for dermatological diagnose. According to the advice from dermatologists, the malignant melanoma is one of the most dangerous skin cancer, and the melanoma lesions have similar visual characteristics to nevus. Therefore, we focus on the melanoma and nevi recognition task on this dataset. We use 12,875 nevi images and 4,522 malignant melanoma images, of which 2,671 images have corresponding lesion mask labels.
- **XJTU-MM:** A skin pathological image dataset collected from the Second Affiliated Hospital of Xi'an Jiaotong University(Xibei Hospital). It contains 9,098 images of RoI regions cropped from the whole slide histopathological images by pathologists, of which 2,170 images are malignant melanoma lesions and 6,928 images are benign nevus. And 726 of them have cell-wise masks labeled by pathologists.

The sample number of three datasets are shown in Table 1. Each dataset is divided into training set, validation set, and test set according to the ratio of 6:2:2, the images of malignant lesions are positive samples and the images of benign lesions are negative samples. Due to not all samples having the corresponding mask label, the shape-biased learning is only optimized when the input images have the corresponding mask labels.

**Table 1:** Number of samples in each dataset.

**Dataset**	**Malignant**	**Benign**	**Total**	Mask label^*^
ISIC-2019	4,522	12,875	17,397	2,671
XJTU-MM	2,170	6,928	9,098	726

^*^Due to not all samples having corresponding mask label, the shape-biased learning is only optimized when the input images have corresponding mask labels.

#### 4.1.2. Evaluation metrics

To quantitatively evaluate the performance of the model, we use accuracy(*Acc*.), precision(*Pre*.), recall(*Rec*.), and F1 score(*F*1) as evaluation metrics. They are calculated by


(13) $$\begin{array}{l} Acc.\text{ =}\frac{TP+TN}{TP+FP+TN+FN},\\ Pre.\;=\frac{TP}{TP+FP},\\ Rec.\text{ =}\frac{TP}{TP+FN},\\ \;F1\;=\frac{2\times Pre.\times Rec.}{Pre.+Rec.}, \end{array}$$

where *TP* (true positive) means the number of samples categorized to positive correctly, *TN* (true negative) means the number of samples categorized to negative correctly, *FP* (false positive) means the number of samples misclassified to malignant, *FN* (false negative) means the number of samples misclassified to negative. Higher accuracy reflects better overall performance of the model on all samples, higher precision means fewer malignant lesions are miss detected, and higher recall means higher sensitivity of the model to malignant lesions, F1 score is the combination of precision and recall. The four metrics provide a comprehensive evaluation of the medical image recognition models.

#### 4.1.3. Implementation

In the proposed STNet-50, ResNet-50 is used as the baseline backbone of texture encoder and shape encoder, the shape feature decoder in the shape-biased stream is constructed using deconvolution operations and referring to the structure of ResNet-18. The texture encoder is pre-trained on ImageNet-1K. We implement the network using pytorch, opencv, scikit-learn and the libraries they depend on based on Python, and train the model on 2 RTX3090-24GB GPUs. All images are resized to 224 × 224, random rotation and random cropping are used for data augmentation. Batch size is set to 64, initial learning rate is set to 5*e*−4, weight decay is set to 1*e*−5, RMSprop (Hinton et al., 2012) is used as the optimization algorithm and the momentum is set to 0.9. The exponential decay factors in asymmetric loss is set to λ_+_ = 1, λ_−_ = 3.

### 4.2. Comparison results

We compared the proposed method with some popular general vision models, including the ResNeSt (Zhang H. et al., 2022), which is the latest iteration of ResNet, and ConvNeXt (Liu et al., 2022), which is regarded as CNN for 2020s. We also added some models designed for specific medical image recognition tasks to the comparative experiment, including DeMAL-CNN (He et al., 2022) for skin lesion classification in dermoscopy images, and MPMR (Zhang D. et al., 2022), which is a multi-scale-feature-based melanoma recognition method in pathological images.

The results are shown in Table 2, which indicate that the proposed STNet outperforms compared algorithms on two datasets and on all evaluation metrics. ConvNeXt series models show generally better performance than ResNeSt-50 on two datasets, which confirms the progress from split-attention block to ConvNet block. DeMAL-CNN shows a similar ability to ConvNeXt on ISIC-2019 dataset, considering that it uses standard ResNet as the backbone, the framework design of DeMAL-CNN has considerable contributions to enhance the dermoscopic image feature representation. MPMR shows better performance than ConvNeXt, which indicates that enhancing multi-scale features is effective in skin pathology image recognition. In addition, in each series of models, the increase in network layers does not bring about significant performance improvements, it is difficult to significantly improve the recognition accuracy of the model simply by increasing the number of layers. Furthermore, in four evaluation metrics, precision and recall are obviously lower than accuracy, which is caused by the sample imbalance of malignant and benign samples. In this case, accuracy cannot comprehensively reflect the performance of the model, it is necessary to add other three metrics.

**Table 2:** Quantitative results of the proposed method and the comparison method on ISIC-2019 and XJTU-MM datasets.

**Dataset**	**Model**	***Acc*.↑**	***Pre*.↑**	***Rec*.↑**	***F*1↑**
ISIC-2019	ResNeSt-50	0.925	0.813	0.923	0.865
	ResNeSt-101	0.927	0.816	0.929	0.869
	ConvNeXt-S	0.949	0.858	0.964	0.908
	ConvNeXt-B	0.957	0.881	0.965	0.921
	DeMAL-50	0.952	0.864	0.967	0.913
	DeMAL-101	0.954	0.878	0.955	0.915
	STNet-50 (ours)	0.967	0.904	0.977	0.939
	STNet-101 (ours)	0.971	0.916	0.978	0.946
XJTU-MM	ResNeSt-50	0.929	0.828	0.885	0.855
	ResNeSt-101	0.933	0.846	0.880	0.863
	ConvNeXt-S	0.945	0.868	0.908	0.887
	ConvNeXt-B	0.946	0.875	0.901	0.888
	MPMR-50	0.958	0.894	0.935	0.914
	MPMR-101	0.961	0.910	0.929	0.919
	STNet-50 (ours)	0.979	0.954	0.959	0.956
	STNet-101 (ours)	0.985	0.963	0.972	0.968

**Figure 6:**
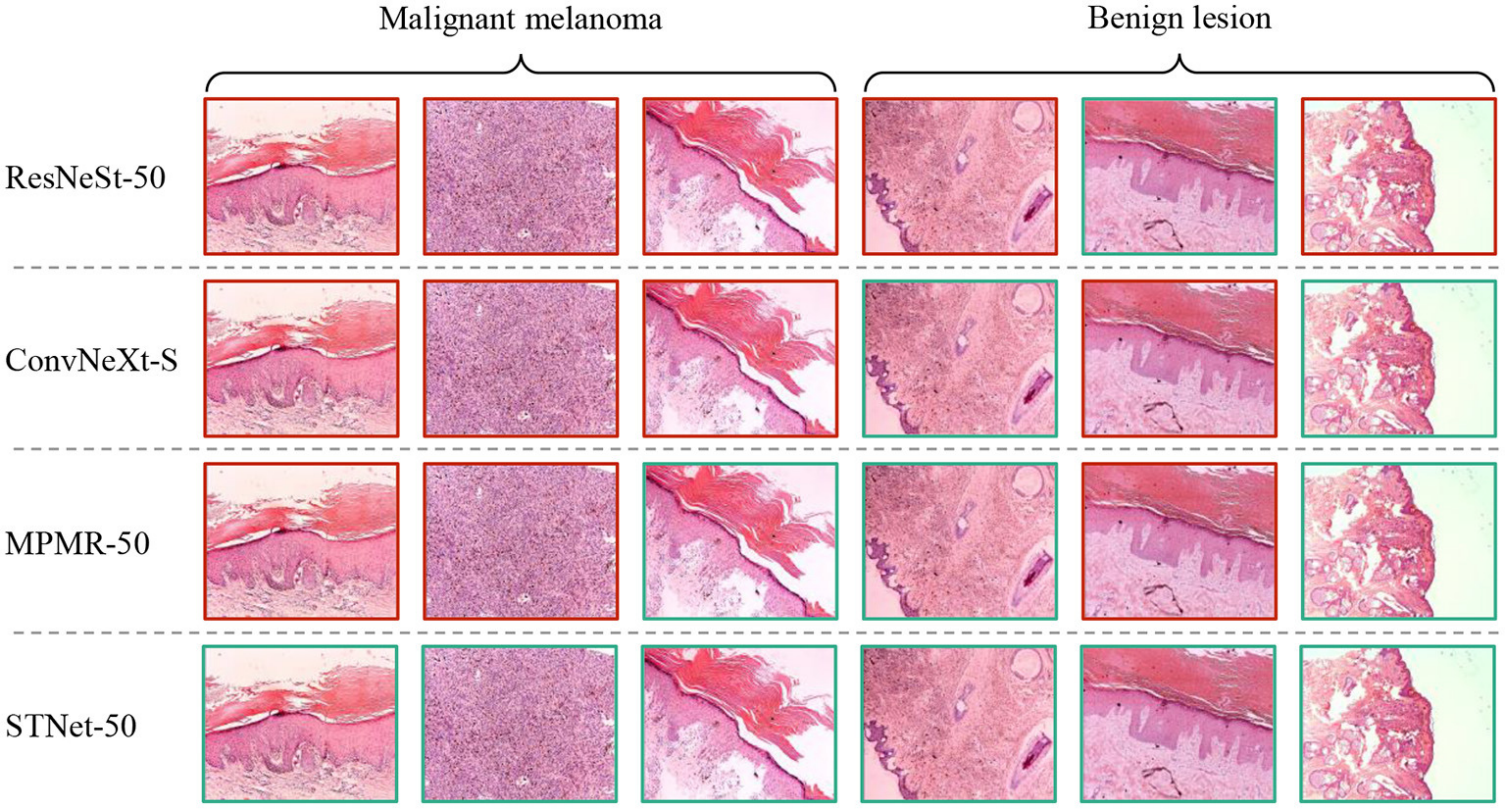
Visualized results of comparative experiment on XJTU-MM dataset. The green boxes mean correctly classified samples, the red boxes mean misclassified samples.

Some difficult samples in the test set of XJTU-MM dataset are visualized and shown in Figure 6, where difficult samples mean the samples near the discriminant hyperplane. According to the results, The proposed STNet-50 correctly recognizes all of these samples. ResNeSt-50, ConvNeXt-S, and MPMR-50 all fail to recognition the first sample and the second sample, which contains rich irregular-shaped features. The fourth sample and the sixth sample have relatively distinct texture features distinct from melanoma, which is relatively easy to identify. The texture and feature joint learning enhances the shape feature representation, and the proposed asymmetric loss guides model to focus on difficult samples, so STNet has advantages on recognizing these difficult samples.

In summary, the results of comparative experiments on ISIC-2019 and XJTU-MM datasets proves the effectiveness of our method.

### 4.3. Ablation analysis

To further study the contribution of each module in our method, we design ablation experiments to analyze the effect of pyramid-grouped convolution(PGC), deformable convolution(DC) and channel-attention-based feature fusion(CAFF) on model performance. we remove all of these modules from the proposed STNet-50 and use it as the baseline model (first row in Table 3). And then PGC, DC and CAFF are rejoined to baseline model one by one (row 2–4 in Table 3). According to the results shown in Table 3, all the three modules bring performance improvement to model, especially in the increase of precision and recall. It indicates that PGC in the texture-biased stream and DC in shape-biased stream can both enhance the feature representation, and CAFF can select features that are more conducive to lesion identification. Additionally, these three modules are portable and can be plugged to other methods.

**Table 3:** Results of ablation analysis of pyramid-grouped convolution(PGC), deformable convolution(DC) and channel-attention-based feature fusion(CAFF) on ISIC-2019 dataset.

**Module**			***Acc*.↑**	***Pre*.↑**	***Rec*.↑**	***F*1↑**

**PGC**	**DC**	**CAFF**				
-	-	-	0.944	0.874	0.915	0.894
✓	-	-	0.951	0.884	0.933	0.908
✓	✓	-	0.959	0.895	0.955	0.924
✓	✓	✓	0.967	0.904	0.977	0.939

**Figure 7:**
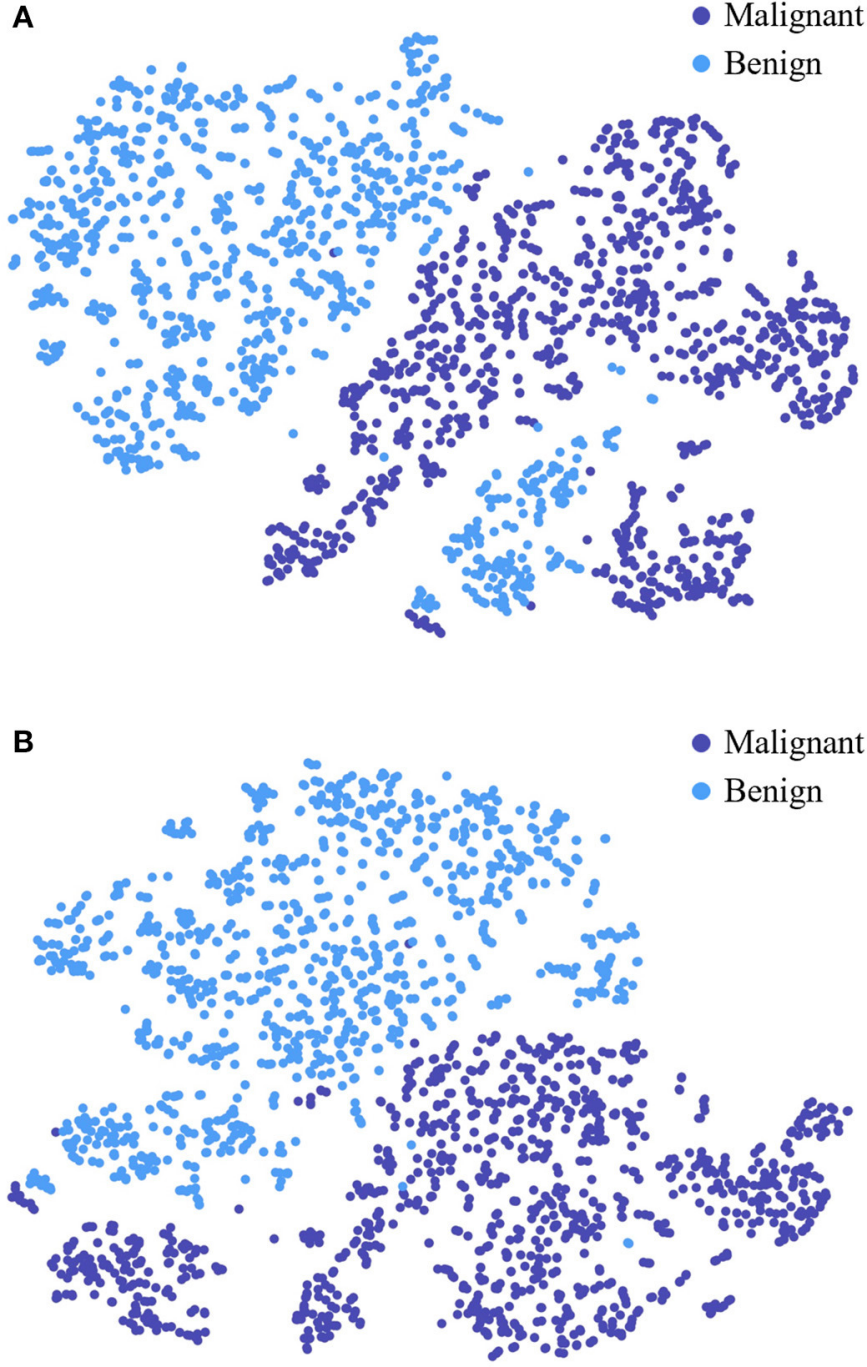
Visualized feature separability analysis through t-SNE. **(A)** Visualized result of STNet-50 without CAFF. **(B)** Visualized result of STNet-50 with CAFF. The feature vectors of STNet-50 with CAFF and STNet-50 without CAFF are transformed to two dimensions, respectively.

To further study the feature selection effect of CAFF in texture and shape feature fusion, we construct STNet-50 with CAFF and without CAFF respectively, and feed 500 malignant samples and 500 benign sample to them, for each sample, the feature vector in front of the classifier is input to t-SNE (Van der Maaten and Hinton, 2008) manifold learning model to study the separability of the extracted features. Through t-SNE, the input feature vectors are transformed into two dimensions and visualized in Figure 7. The comparison of Figures 7A, B show that the feature vector of the model with CAFF is more separable, which is conductive to classification. The results indicate that the introduction of CAFF module is effective to select features relevant to lesion recognition.

Due to the available data is limited, to verify performance of the proposed model more rigorously, we conducted five-fold cross-validation on both ISIC-2019 and XJTU-MM datasets. Each dataset was divided into five mutually exclusive parts, with four used for training the STNet-50 model and one remaining part used for testing. Because of the sample imbalance problem, we use *F*1 score as the evaluation metric. The cross-validation results are shown in Table 4, STNet-50 shows consistent performance in each fold of the cross-validation, which proves the stability and reliability of the results.

**Table 4:** Five-fold cross-validation results of the proposed STNet-50 model on ISIC-2019 and XJTU-MM datasets.

**Datasets**	***F***1 **score** ↑				
	**Fold 1**	**Fold 2**	**Fold 3**	**Fold 4**	**Fold 5**
ISIC-2019	0.939	0.930	0.932	0.939	0.935
XJTU-MM	0.956	0.953	0.955	0.953	0.952

### 4.4. Discussion on shape and texture joint learning framework

We propose the two-stream network for texture and shape joint learning, compared to single-stream network, an extra shape feature encoder is introduced. To analyze the contributions to performance improvements are provided by texture and shape joint learning or just the extra feature encoder, three control group models are designed for the comparative experiment. The first model uses the texture encoder only for feature extraction. The second model cascades the segmentation network and the classification network in the proposed method, the segmented lesion is used as the input of the classification network. The third model is constructed by removing the feature decoder of the shape-biased stream in our method, which is a two-stream network but without shape and texture joint learning. ISIC-2019 dataset is used for this experiment, the results are shown in Table 5, compared to the single-stream model, the cascade classification and segmentation model does not show obvious performance improvement and even have a performance drop on recall. It means that when the lesion mask labels are not sufficient, cascading the segmentation network and the classification network has limitation in solving weak shape representation problems. Two-stream network with joint learning shows better performance than that without joint learning, it indicates that the performance improvement of the proposed method is not simply brought by the extra shape feature encoder but by shape and texture joint learning, which proves the effectiveness of our method.

**Table 5:** Experiments of discussion on shape and texture joint learning.

**Backbone layers**	**Structure**	***Acc*.↑**	***Pre*.↑**	***Rec*.↑**	***F*1↑**
50	Single-stream^a^	0.950	0.872	0.945	0.907
	Cascade Cls. and Seg.^b^	0.950	0.886	0.928	0.907
	Two-stream without joint learning^c^	0.960	0.909	0.939	0.924
	Two-stream with joint learning^d^	0.967	0.904	0.977	0.939
101	Single-stream^a^	0.952	0.877	0.950	0.912
	Cascade Cls. and Seg.^b^	0.955	0.888	0.945	0.916
	Two-stream without joint learning^c^	0.961	0.911	0.944	0.927
	Two-stream with joint learning^d^	0.971	0.916	0.978	0.946

^a^Single-stream: only use the texture encoder in the proposed method for feature extraction.

^b^Cascade Cls. and Seg.: cascading segmentation network in front of classification network.

^c^Two-stream without joint learning: removing the feature decoder in the shape-biased stream of our method.

^d^Two-stream with joint learning: the proposed framework.

### 4.5. Discussion on parameters of asymmetric loss

**Figure 8:**
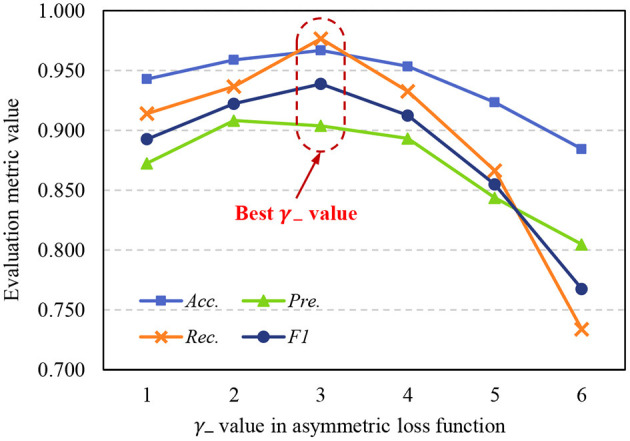
Variation of evaluation metrics with γ_−_ when γ_+_ = 1. *Acc*., accuracy; *Pre*., precision; *Rec*., recall; *F*1, F1 score.

The asymmetric loss function in the proposed method is designed to address the sample imbalance problem, we use exponential decay factors γ_+_ and γ_−_ to adjust the attention of the model to positive and negative classes. Due to in medical image datasets, malignant samples are usually much fewer than benign samples, γ_−_ should achieve a stronger decay effect, so γ_+_ < γ−. To further study the effects of γ_+_ and γ_−_ to model performance, we set γ_+_ = 1, and use different γ_−_ to train the STNet-50 on ISIC-2019 dataset, the test results are shown in Figure 8. Despite the model achieving the highest *Pre*. value When γ_−_ = 2, taking into account the four metrics, the model has the best performance when γ_−_ = 3. When γ_−_ is too small, exponential decay is not enough to eliminate the impacts of sample imbalance. When γ_−_ is too large, the effect of exponential decay is so strong that the model tends to ignore negative samples, and the performance of the model drops significantly. According to the results in Figure 8, choosing an appropriate value of the exponential decay factor is important to train a good-performance model.

## 5. Conclusion

In this paper, we propose the two-stream shape and texture joint learning network to address the weak shape feature representation problem of existing medical image recognition methods. According to the experiments on ISIC-2019 and XJTU-MM datasets, the proposed two-stream network is an effective method to combine texture and shape features. In addition, the proposed pyramid-grouped convolution enhances the texture feature representation, and deformable convolution enhances the shape feature representation. Furthermore, the channel-attention-based feature fusion module effectively eliminates redundant information and selects essential features. The asymmetric loss function addresses the problem of sample imbalance. The proposed method improves the model performance on shape-relied medical image recognition tasks, and provides support for computer-aided imaging diagnosis. Additionally, in our method, to enhance shape feature representation, an extra feature encoder is introduced, which increase the computation requirements, although the computation. Although inference speed is not the most critical concern in medical image analysis, we aim to enhance shape and texture feature representation by avoiding the use of additional encoders in future work, enhancing shape feature representation and texture feature representation within a single encoder.

## Data availability statement

The raw data supporting the conclusions of this article will be made available by the authors, without undue reservation.

## Author contributions

XW provided some ideas for this work. HH designed and implemented the models, ran the experiments, and wrote the manuscript. MenX analyzed the experimental data and visualized the results. SL helped write a part of the manuscript. DZ helped analyze the data and checked the manuscript writing. SD was in charge of project management. MeiX helps manage the project and provided advice for data analysis. All authors contributed to the article and approved the submitted version.
